# Actin and Myosin-Dependent Localization of mRNA to Dendrites

**DOI:** 10.1371/journal.pone.0092349

**Published:** 2014-03-17

**Authors:** Varuzhan Balasanyan, Don B. Arnold

**Affiliations:** Department of Biology, Program in Molecular and Computational Biology, University of Southern California, Los Angeles, California, United States of America; Western University of Health Sciences, United States of America

## Abstract

The localization of mRNAs within axons and dendrites allows neurons to manipulate protein levels in a time and location dependent manner and is essential for processes such as synaptic plasticity and axon guidance. However, an essential step in the process of mRNA localization, the decision to traffic to dendrites and/or axons, remains poorly understood. Here we show that Myosin Va and actin filaments are necessary for the dendritic localization of the mRNA binding protein Staufen 1 and of mRNA encoding the microtubule binding protein Map2. Blocking the function or expression of Myosin Va or depolymerizing actin filaments leads to localization of Staufen 1 and of Map2 mRNA in both axons and dendrites. Furthermore, interaction with Myosin Va plays an instructive role in the dendritic localization of Hermes 1, an RNA binding protein. Wild-type Hermes 1 localizes to both axons and dendrites, whereas Hermes 1 fused with a Myosin Va binding peptide, localizes specifically to dendrites. Thus, our results suggest that targeting of mRNAs to the dendrites is mediated by a mechanism that is dependent on actin and Myosin Va.

## Introduction

Localization of mRNAs to dendrites and axons has been implicated in many processes, including synaptic plasticity [1], [2] and axon guidance [3], [4], and dysregulation of dendritic translation has been linked to neurological conditions such as autism [5], [6] and mental retardation [7]. The mechanisms underlying localization of mRNAs to axons and dendrites, however, remain poorly understood. Studies in non-neuronal cells have identified the major molecular components involved in subcellular localization of mRNAs. Within the 3′ untranslated regions of mRNAs regions known as zip codes are necessary and sufficient for subcellular localization [8]–[10]. Zip codes bind to RNA binding proteins, which form bridges between mRNAs and transport machinery [11], [12]. Subcellular localization of mRNAs also depends on microtubules and on the motor proteins kinesin and dynein. For instance, in oocytes of D. melanogaster posterior localization of Oskar mRNA and its binding protein Staufen is mediated by Kinesin I, and anterior localization of mRNA encoding Bicoid and Gurkin is dependent on Dynein [13], [14].

In neurons zip codes and their associated mRNA binding proteins mediate transport to the dendrites and/or axons [15]–[20]. In addition, the kinesins Kif5 and Kif3A associate with dendritic ribonucleoprotein particles (RNPs), suggesting that they mediate RNP transport [21], [22]. Myosin motors are also involved in mRNA transport and localization. Myosin Va regulates axonal movements of Zipcode Binding Protein [23] and is necessary for localizing the Nd1-L mRNA and its binding protein, Translocated in Liposarcoma (TLS), to dendritic spines [24]. Myosin Va has also been implicated in the localization to dendrites of transmembrane proteins such as ion channels, receptors and transporters [25]. However, since RNPs are not membrane delimited and do not associate with transmembrane proteins, it is not known whether Myosin Va might play a similar role in the dendritic localization of mRNA.

Here we test the hypothesis that Myosin Va and actin contribute to the dendritic localization of mRNAs. We find that in dissociated rat cortical neurons disrupting actin filaments, expressing a dominant negative variant of Myosin Va, or knocking down Myosin Va disrupts the somatodendritic localization of Staufen 1, an mRNA binding protein. Similarly, expressing a Myosin Va dominant negative construct or disrupting actin filaments causes Map2 mRNA, whose expression is normally restricted to the soma and dendrites [26], to be present in the axon. Finally, Hermes 1, an RNA binding protein that is present in both axons and dendrites [27], localizes to the somatodendritic compartment when fused to a Myosin Va binding domain. Thus, our results are consistent with Myosin Va and actin causing the expression of mRNAs to be restricted to the somatodendritic compartment.

## Materials and Methods

### Ethics Statement

Experimental protocols were conducted according to the US National Institutes of Health guidelines for animal research and were approved by the Institutional Animal Care and Use Committee at the University of Southern California.

### cDNA constructs

EGFP-Stau1 and EGFP-Hermes1 were generated by inserting the sequence encoding rat Stau1 (NP_445888: 2-495 aa), or the sequence encoding rat Hermes1 (NP_001258173.1: 1-220 aa), downstream of the sequence encoding EGFP in GW-EGFP, an expression vector. EGFP-Hermes1-MBD was created by inserting the sequence encoding Melanophilin residues 176–201 downstream of Hermes1. HA-dnMyoVa was made by inserting the sequence encoding amino acids 1006–1828 of rat Myosin Va (AB_035736.1) upstream of the sequence encoding an HA tag. A partial gene sequence of rat Map2 gene (NM_013066.1: 2420-3080 nt) was subcloned into the pBluscript II KS+ vector using the XhoI and XbaII restriction sites in both direct and inverted orientations in order to synthesize anti-sense and sense strands, respectively, using T3 RNA polymerase (the T3 RNA polymerase promoter were positioned downstream to XbaII site.)

### Dissociated Culture and Transfection

Dissociated cultures of cortical neurons from rats of both sexes were prepared following the Banker method. Briefly, cells from the rat embryonic cortex were isolated at embryonic day E18 (from 8 pups), dissociated in HBS-Hanks buffer, and plated on poly-D-lysine and Laminin pre-treated glass coverslips (22 mm×22 mm×0.17 mm, Thermofisher, Waltham, MA, USA) in 6-well tissue culture plates at a density of 2–5×10^4^ cells per well. Wells contained neurobasal medium (Invitrogen, Carlsbad, CA, USA) supplemented with 10 ml/L Glutamax (Invitrogen, Carlsbad, CA, USA), 1 μg/ml gentamicin (Invitrogen, Carlsbad, CA, USA), 20 ml/L B-27 supplement (Invitrogen, Carlsbad, CA, USA) and 50 ml/L fetal bovine serum (Invitrogen, Carlsbad, CA, USA). The culture medium was changed once on the day of plating to reduce the concentration of FBS from 5% (attachment condition) to 1.5% (growth condition) in the final medium, and once every week thereafter.

Neurons were transfected at 12–18 d *in vitro* by Ca^2+^ phosphate. Briefly, a mixture of different plasmid DNAs (comprising a total of 8 μg of DNA) encoding the genes of interest were mixed in sterile deionized water (up to 87.4 μl). 12.6 μl of 2 mM CaCl_2_ was added to the mixture to neutralize the negative charge of the plasmid DNA. This mixture of DNA and CaCl_2_ (100 μl total) was added drop-wise into 100 μl of 2X HBS, pH = 7.04. Crystals were allowed to form for 20 min at room temperature and the resulting mixture of the DNA, CaCl_2_, and 2X HBS (200 μl total) was added drop-wise on top of the neurons in the wells. Crystals were allowed to form efficiently for approximately 2–4 h (incubation time depended on the age of the neurons). After the allotted incubation time, the crystals were washed from the neurons by NaCl-HEPES buffer, pH = 7.2, for a few seconds, and the neurons on coverslips were placed into 50% conditioned medium/50% fresh medium (4 ml total) that had been pre-warmed in the tissue culture incubator. The expression of exogenous constructs was checked the following day under an epiflourescence microscope before proceeding to immunostaining or FISH protocols.

### Immunocytochemistry

Following the expression of exogenous proteins overnight, the neurons were fixed in 4% paraformaldehyde in 1X PBS for 5 min and washed three times with 1X PBS for 5 min. The cells were then permeabilized and incubated in a blocking buffer (1% Bovine Serum Albumin, 5% Normal Goat Serum, 0.1% Triton X-100 in 1X PBS) for 30 minutes. Cells were incubated in combinations of the following primary antibodies at the following dilutions: chicken GFP (Millipore, Bellerica, MA, USA), 1∶15000; mouse AnkG (Neuromab, Davis, CA, USA), 1∶500; mouse HA (Covance, Princeton, NJ, USA), 1∶500; rabbit AnkG (Santa Cruz Biotechnology, Santa Cruz, CA, USA), 1∶1000; rabbit Myosin Va (Sigma, St. Louis, MO, USA), 1∶500; mouse Tubulin (Sigma, St. Louis, MO, USA), 1∶500; rabbit Stau1 (a generous gift from Dr. M. Kiebler), 1∶500 overnight. After incubation with the Alexa fluorophore-conjugated secondary antibodies (all secondary antibodies were used in 1∶2000 dilution (Invitrogen, Carlsbad, CA, USA)) for 30 hours, the neurons were mounted on glass slides using Fluoromount G (Electron Microscopy Sciences, Hatfield, PA, USA).

### Riboprobe Synthesis

Plasmid constructs encoding Map2 sense and antisense probes were linearized using the XhoI site. T3 RNA polymerase (Promega, Fitchburg, WI, USA) was used to synthesize sense and anti-sense riboprobes from two linearized DNA templates in the presence of the DIG-UTP (DIG RNA labeling mix, Invitrogen, Carlsbad, CA, USA). After 2 h of *in vitro* synthesis, RNAse-free DNAse (Promega, Fitchburg, WI, USA) was added to the transcription reaction mixture and incubated for an additional 15 min at 37°C to destroy the DNA template. The synthesized riboprobes were then precipitated with LiCl to remove the unincorporated nucleotides, and the resulting pellets were re-suspended in nuclease-free water (Promega, Fitchburg, WI, USA).

### Fluorescent In Situ Hybridization

Fluorescent In Situ Hybridization (FISH) technique was performed following the protocol from Dr. K. Martin's lab [28]. 17 DIV rat cortical neurons on glass coverslips were fixed with 4% paraformaldehyde in 1X DEPC PBS (PBS made with DEPC-treated, autoclaved H_2_0) for 10 min, and followed by three 10 min washes with 1X DEPC PBS and two 10 min washes with 1X PBS containing 0.1% active DEPC. After permeabilization with 0.5% Triton X-100 containing 1X DEPC PBS, the cells were washed 3X each for 10 min with 1X DEPC PBS. To minimize nonspecific riboprobe hybridization, the neurons were incubated in pre-hybridization buffer (50% formamide (Thermofisher, Waltham, MA, USA), 5X SSC (Sigma, St. Louis, MO, USA), 40 μg/mL salmon sperm DNA (Invitrogen, Carlsbad, CA, USA), 2% blocking reagent (Roche, Indianapolis, IN, USA)) in a humidified incubator at a hybridization temperature (58–60°C) that was defined by the melting temperature of the given riboprobe. After pre-hybridization, the appropriate amounts of sense and antisense riboprobes were added to the hybridization buffer, denatured at 72°C for 15 min and put on ice immediately afterwards for 5 min. The probes were hybridized to tissue samples for 48 h at 59°C in a humidified chamber. After hybridization, the solution containing the probes was removed from the coverslips, and the cells were washed twice for 5 min with 2X SSC at room temperature, followed by a 1 h wash with 2X SSC/50% formamide at 50°C and a 5 min wash with 1X DEPC in PBS. To reduce the endogenous peroxidase activity, the cells were treated with 3% H_2_O_2_ diluted in 1X DEPC/PBS for 5 min, followed by three 5 min washes with Buffer 1 (100 mM Tris, 150 mM NaCl, pH = 7.5). In order to perform immunostaining on cell samples that had already been hybridized, neurons were treated with the blocking buffer (Buffer 1 + 10% normal goat serum) for 30 min at room temperature. The neurons were then incubated with primary antibodies diluted in blocking buffer for 1 h at room temperature. The following primary antibodies were used: Anti-DIG POD antibody (Roche, Indianapolis, IN, USA), diluted 1∶500; chicken GFP antibody (Thermofisher, Waltham, MA, USA), diluted 1∶1000; mouse HA (Covance, Princeton, NJ, USA), diluted 1∶500. To remove unbound antibodies, the cells were washed 3X for 5 min each with TNT Buffer (Buffer 1 + 0.3% Triton X-100) and equilibrated by incubation in Buffer 1. To detect riboprobe that was hybridized to its target mRNA signal amplification using Cy3-labeled tyramide was used (Perkin Elmer, Waltham, MA, USA). To detect the immunostained proteins Alexa fluorophore-conjugated secondary antibodies (Invitrogen, Carlsbad, CA, USA) were used.

### siRNA experiments

Five different candidate siRNA sequences against the rat Myosin Va gene were chosen from the published literature and checked for specificity using BLAST. Based on those sequences, hairpins were designed in an antisense-*loop*-sense-*TTTTT* orientation and subcloned downstream of the U6 promoter into shRNA-expressing vectors (Origene, Rockville, MD, USA). Of five different siRNAs, only one (5′-GTCAATCAGGCTCTCCATTCTGCTGTCAA-3′, nt 1255 –1283 of Myosin Va) down-regulated expression of endogenous Myosin Va protein efficiently in cortical neurons within 3-4 days. To create a variant that was impervious to siRNA (Myosin Va Mut1275) for rescue experiments, the mutation C1275A was made in the Myosin Va gene. As a negative control for these siRNA experiments we used a scrambled siRNA that is not complementary to any known rat mRNA.

### Image Capture and Analysis

All images were taken on an Olympus IX71 inverted microscope using a 60×/1.20W objective (UplanApo/IR, Japan) with the maximum range of pixel saturation. Cells were chosen for imaging that had good overall morphology and clearly definable axons that did not overlap with dendrites. Axons were identified either by staining against endogenous Ankyrin G or as: (i) the longest process. (ii) having a non-tapered shape. (iii) having no spines. Both the capture and analysis of images (ImageJ) were performed without previous knowledge of the condition.

To quantify the polarized distribution of mRNA binding proteins we calculated the axon to dendrite ratio (ADR), defined as the ratio of the mean fluorescence signal/pixel in the distal axon (40 μm away from the axon base) to the same measurement in the entire dendritic compartment. In general, nonspecifically localized proteins had an ADR of greater than 0.4, whereas dendritically localized proteins had a ratio less than 0.2. All errors were expressed as the standard error of the mean. The significance of differences between ADRs for different pairwise conditions was assessed using Wilcoxon-Mann-Whitney statistical test for nonparametric data. In cases when more than two groups were compared the Kruskal-Wallis test was used.

## Results

### Myosin Va is necessary for somatodendritic localization of exogenous Staufen 1

**Figure 1:**
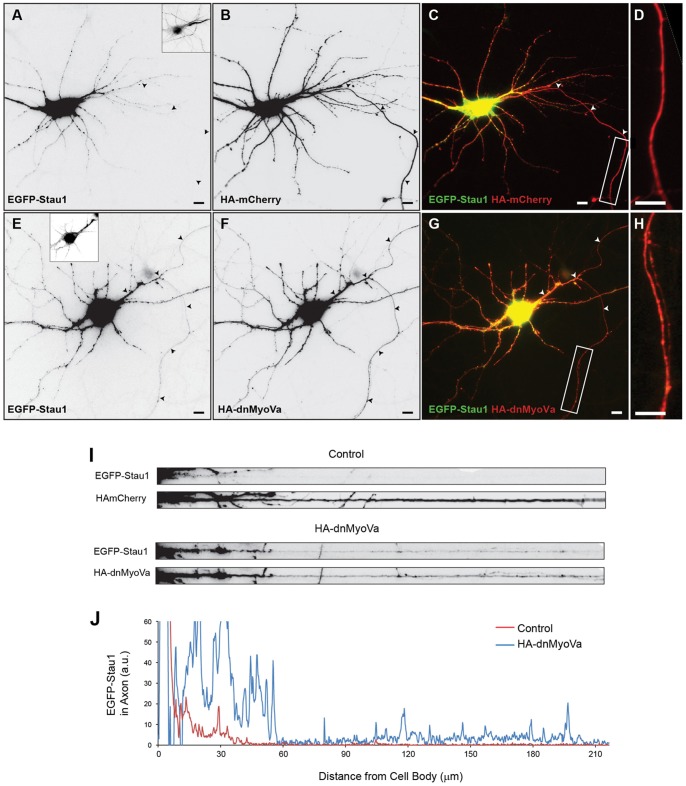
Myosin Va is necessary for somatodentritic targeting of EGFP-Stau1. **A.** Following expression in a cortical neuron in dissociated culture EGFP-Stau1 is present in the soma and dendrites, but not in the axon. **B**. Co-expressed HA-mCherry localizes to the axon as well as to the soma and dendrites in the same cell as in (A). **C**. Merge of HA-mCherry (red) and EGFP-Stau1 (green). **D.** High power image of boxed region from (**C**). **E**. EGFP-Stau1 is present in the axon as well as in the soma and dendrites when co-expressed with HA-dnMyoVa (**F**), a dominant-negative variant of Myosin Va. Arrowheads point to the axon. Scale bars represent 10 μm. **G**. Merge of HA-dnMyoVa (red) and EGFP-Stau1 (green). **H**. High power image of boxed region from (G). **I**. A straightened axon from the neuron in (A-D) co-expressing HA-mCherry (upper panel) shows a relative absence of EGFP-Stau1 staining. In contrast, a straightened axon from the neuron in (E-H) expressing HA-dnMyoVa (lower panel) shows EGFP-Stau1 staining. **J**. EGFP-Stau1 staining vs. distance in the distal axon for the neuron in (A-D; red) and the neuron in (E-H; blue).

Interaction with Myosin Va mediates somatodendritic localization of ion channels, transporters and receptors by preventing them from entering the axon [29]. Accordingly, we asked whether it might play a similar role in the localization of dendritic mRNAs. As a marker for RNPs we chose to examine the localization of Staufen 1, an mRNA binding protein that is localized to dendrites [18], [30], [31]. Previously it has been shown that Myosin Va and Staufen 1 are found in the same RNP particles following purification from mouse brain lysate [32]. Accordingly, we investigated whether Myosin Va might act to mediate localization of Staufen 1 to dendrites. To do so, we expressed EGFP-Staufen 1 (EGFP-Stau1) in dissociated cultures of cortical neurons at 12–17 days *in vitro* using calcium phosphate precipitation (see methods). Following expression for 16–18 hours, EGFP-Stau1 was found to be present in the soma and dendrites, but absent from the axon (Fig. 1A-D). Note that the axon was identified using Ankyrin G staining of the axon initial segment. In order to block Myosin Va function, we expressed a dominant negative variant consisting of its globular tail domain, tagged with a Hemaglutinin tag (HA-dnMyoVa) [33]. Co-expression of EGFP-Stau1 with HA-dnMyoVa resulted in the expression of EGFP-Stau1 in axons as well as in dendrites (Fig. 1E-H). Images showing expression of EGFP-Stau1 in a straightened axon co-expressing HA-dnMyoVa show a clear increase of EGFP-Stau1 when compared to a similar cell co-expressing HA-mCherry (Fig. 1I). Furthermore, plots of the intensity of EGFP-Stau1 in the axons (Fig. 1J) show an increase in expression in the cell expressing HA-dnMyoVa versus in the control cell. Quantitation of the localization of EGFP-Stau1 confirms these qualitative findings. The axon to dendrite ratio (ADR), a comparison of the relative amount of EGFP-Stau1 in the two compartments (see methods), is 0.08 +/− 0.01, n = 12 for control cells, whereas in cells co-expressing HA-dnMyoVa, ADR_EGFP-Stau1, HA-dnMyoVa_ = 0.56 +/− 0.11, n = 13, a significant difference (p<0.0001, Wilcoxon Mann Whitney test). These results are consistent with Myosin Va being necessary for the localization of EGFP-Stau1 specifically to the somatodendritic compartment.

### Myosin Va is necessary for somatodendritic localization of endogenous Staufen 1

**Figure 2:**
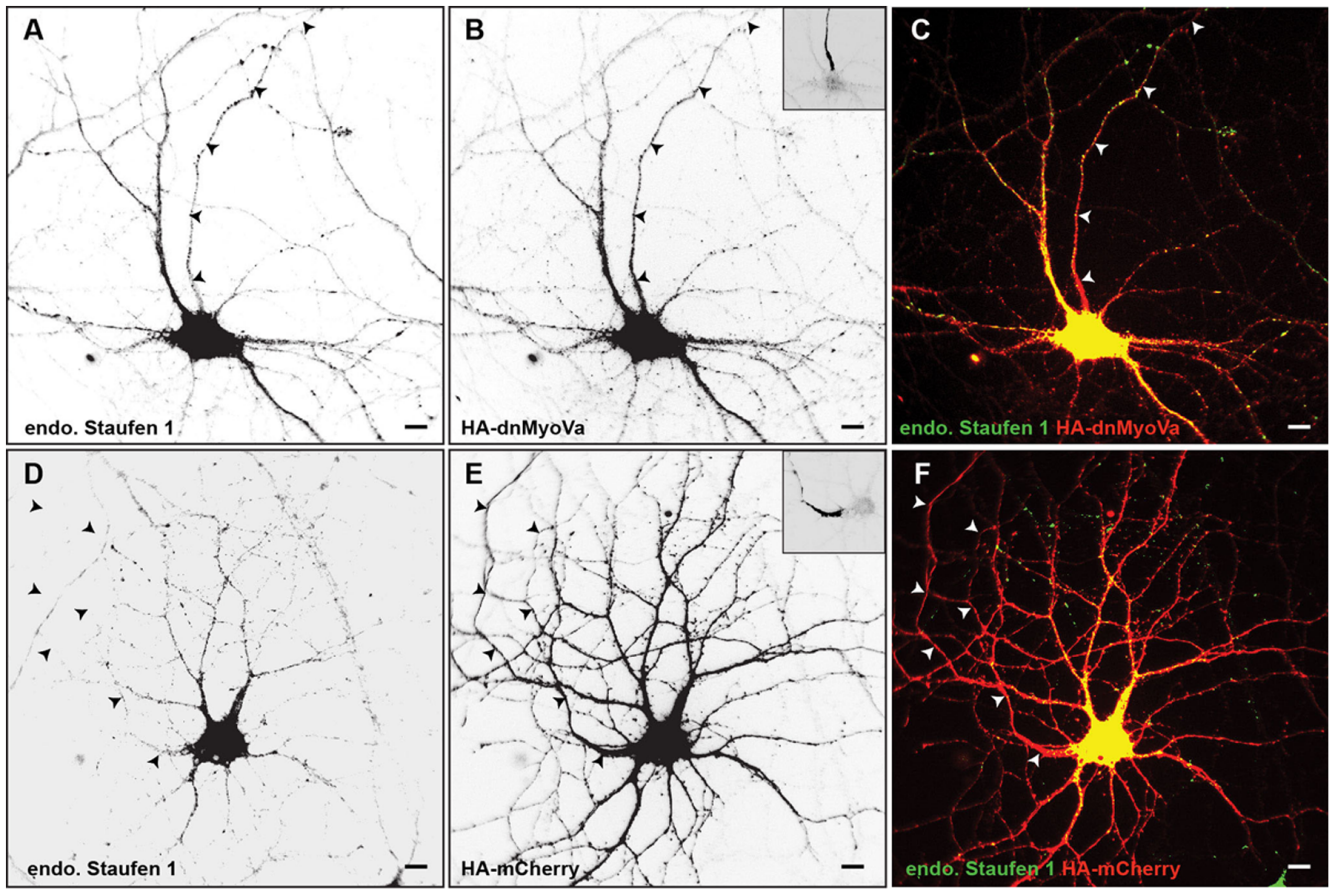
Myosin Va is necessary for somatodentritic targeting of endogenous Staufen1. **A**. Endogenous Staufen 1 is present in the axon as well as in the soma and dendrites when co-expressed with HA-dnMyoVa (**B**), a dominant-negative variant of Myosin Va. **C**. Merge of endogenous Staufen 1 (green) and HA-dnMyoVa (red). **D**. Endogenous Staufen 1 is completely absent from the axonal compartment, but is expressed in the somatodendritic compartment of cortical neurons, expressing HA-mCherry (**E**). **F**. Merge of endogenous Staufen 1 (green) and HA-mCherry (red). Arrowheads point to the axon. Scale bars represent 10 μm. Insets show staining for Ankyrin G, a marker for the axon initial segment.

To corroborate the previous results obtained with EGFP-Stau1, we investigated whether Myosin Va is necessary for the localization of endogenous Staufen 1. In cortical neurons in dissociated culture expressing HA-dnMyoVa for 48 hours immunostaining revealed that endogenous Staufen 1 is expressed in both axons and dendrites (Fig. 2A-C; ADR_endStau1, HA-dnMyoVa_ = 0.59 +/- 0.11, n = 12). In contrast, in cells expressing HA-mCherry endogenous Staufen 1 localized specifically to the dendrites (Fig. 2D-F; ADR_endStau1, HAmCherry_  = 0.13 +/− 0.03, n = 10). The difference in respective ADRs was significant (p<0.001, Wilcoxon). Thus, our results are consistent with Mysoin Va being necessary for the dendritic localization of both exogenously expressed, and endogenous, Staufen 1.

### Myosin Va is necessary for somatodendritic localization of Map2 mRNA

**Figure 3:**
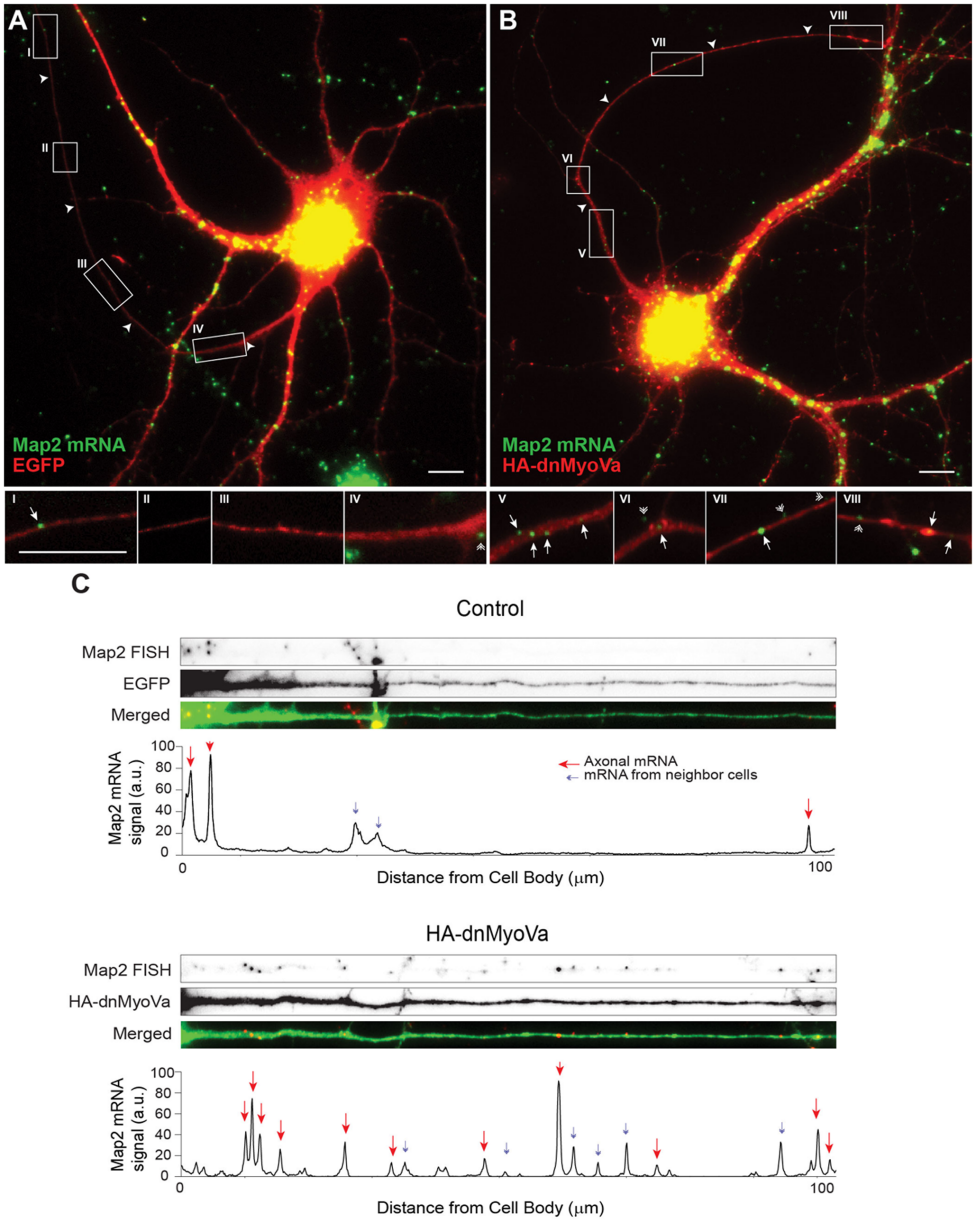
Myosin Va is necessary for somatodendritic targeting of endogenous Map2 mRNA. **A**. Map2 mRNA labeled by fluorescent in situ hybridization (green) in a cortical neuron in dissociated culture expressing EGFP (red). Map2 mRNA is present in the somatodendritic compartment, but largely absent from the axon (arrowheads). Insets I-IV are high power images corresponding to boxes drawn in (A). **B**. In a cortical neuron expressing HA-dnMyoVa, staining for endogenous Map2 mRNA is present in the axon as well as in the somatodendritic compartment. Insets V-VIII are high power images corresponding to boxes in (B). Arrowheads point to axons, arrows to Map2 mRNAs that are present in the axon, double arrowheads to Map2 mRNAs from neighboring untransfected neurons. Scale bar, 10 μm. **C**. Signal intensity plots of Map2 mRNA staining in straightened axons of the control neuron in (A) and the neuron expressing HA-dnMyoVa in (B). The presence of HA-dnMyoVa is correlated with an increase in the amount of Map2 mRNA in the axon. Red arrows point to Map2 mRNA puncta in the axon. Blue arrows point to Map2 mRNA puncta from neighboring neurons.

To further investigate a possible role for Myosin Va in the localization of RNPs, we used fluorescent in situ hybridization (FISH) to directly label mRNA within the dendrites of cortical neurons. We examined Map2 mRNA, because it is abundantly expressed, has a well-defined somatodendritic localization, and has been shown to interact with Staufen 1 [26], [34]. When FISH was performed on dissociated cortical neurons using anti-Map2 probes, we found that labeled puncta were abundant in the soma and dendrites, but were almost completely absent from the axon (Fig. 3A) of control cells. In contrast, cortical neurons expressing HA-dnMyoVa for 48 hours showed Map2-labeled puncta in the axon (Fig. 3B). Note that labeling of similar cultures with the sense probe produced negligible labeling that colocalized with neuronal processes, indicating that puncta that colocalize with processes are likely not due to background labeling (Fig. S1). Images of Map2-labeled puncta in straightened axons as well as plots of the signal produced by the label (Fig. 3C) highlight the dramatic difference between control cells, where there are few, if any, puncta in the distal axon and cells expressing HA-dnMyoVa where multiple puncta are present in the distal axon. ADR_ Map2, Control_ = 0.12 +/− 0.02, n = 16, whereas ADR_Map2, HA-dnMyoVa_ = 0.50 +/− 0.05, n = 19, a difference that is statistically significant (p<0.001, Wilcoxon). Thus, both mRNA and an mRNA-associated protein are mislocalized when Myosin Va function is blocked, suggesting that Myosin Va is necessary for confining dendritic RNPs to the somatodendritic compartment.

**Figure 4:**
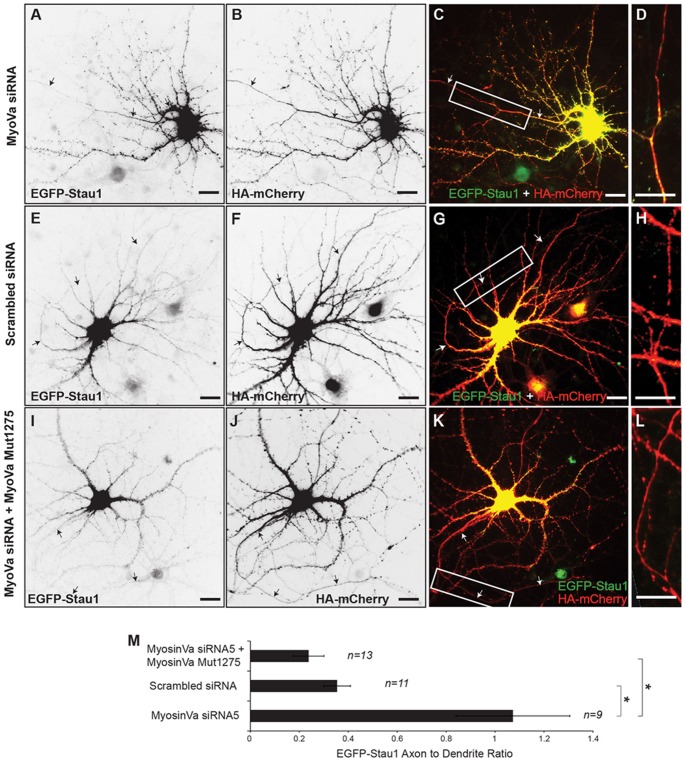
Knockdown of Myosin Va disrupts somatodendritic targeting of EGFP-Stau1. **A**. EGFP-Stau1 localizes to both the axon and the somatodendritic compartment in a cortical neuron transfected with MyoVa siRNA and HA-mCherry (**B)**. **C**. Merge of EGFP-Stau1 (green) and HA-mCherry (red). **D**. High power image of boxed area in (C). **E**. EGFP-Stau1 localizes in the soma and dendrites, but not in the axon, of a neuron expressing scrambled siRNA and HA-mCherry (**F)**. **G**. Merge of EGFP-Stau1 (green) and HA-mCherry (red). **H**. High power image of boxed area in (G). **I**. EGFP-Stau1 localizes in the soma and dendrites, but not in the axon, of a neuron expressing MyoVa siRNA as well as MyoVa Mut1275 and HA-mCherry (**J**). **K**. Merge of EGFP-Stau1 (green) and HA-mCherry (red). **L**. High power image of boxed area in (K). Scale bar, 10 μm. Note that this result indicates that blocking of somatodendritic localization of EGFP-Stau1 by MyoVa siRNA is not due to off-target effects. **M**. Axon to dendrite ratio of EGFP-Stau1 in neuron expressing MyoVa siRNA is significantly different from those in neurons expressing either scrambled siRNA or MyoVa siRNA and MyoVa Mut1275. * indicates p<0.001 (Kruskal-Wallis).

To corroborate the experiments done with HA-dnMyoVa, we examined the localization of EGFP-Stau1 following knockdown of Myosin Va expression using siRNA. We found that expression of siRNA reduced expression of endogenous Myosin Va by 70-80% in cortical neurons after 4 days (Fig. S2). In cortical neurons expressing siRNA against Myosin Va co-expressed EGFP-Stau1 localized in both axons and dendrites after 4 days of expression (Fig. 4A-D, ADR_EGFP-Stau1_, _MyoVa siRNA_ = 1.1 +/− 0.2, n = 9). In contrast, in neurons co-expressing scrambled siRNA EGFP-Stau1 was localized preferentially to the somatodendritic compartment (Fig. 4E-H; ADR _EGFP-Stau1,_ _siRNA scrambled_ = 0.35 +/− 0.05, n = 11). To determine whether the effect of siRNA against Myosin Va on localization of EGFP-Stau1 was due to off-target effects we coexpressed EGFP-Stau1 and Myosin Va siRNA along with a Myosin Va cDNA that was mutated so that it is impervious to siRNA, but has the same amino acid sequence as wild-type Myosin Va, in cortical neurons (MyoVa Mut1275). Under these circumstances EGFP-Stau1 localized specifically to the somatodendritic compartment (Fig. 4I-L, ADR _EGFP-Stau1, MyoVa_ _siRNA + MyoVaMut_ = 0.24 +/− 0.06, n = 13). ADR_EGFP-Stau1_, _MyoVa siRNA_ is significantly different from ADR _EGFP-Stau1,_ _siRNA scrambled_ and ADR _EGFP-Stau1, MyoVa_ _siRNA + MyoVaMut_ (Fig. 4M, p<0.001, Kruskal-Wallis). Thus, the effects of siRNA against Myosin Va are likely specific and corroborate the findings from dominant negative experiments that Myosin Va is necessary for localization of EGFP-Stau1 to dendrites. Note that in these experiments, EGFP-Stau1 was expressed for four days as opposed to the experiments in Fig. 1, where it was expressed for two days. Longer expression appears to be correlated with higher ADRs regardless of what constructs were co-expressed with EGFP-Stau1. Thus, experiments in Fig. 4 cannot be directly compared with those where EGFP-Stau1 was expressed for a shorter time.

### Actin filaments are necessary for localization of exogenous Staufen 1 and Map2 mRNA

**Figure 5:**
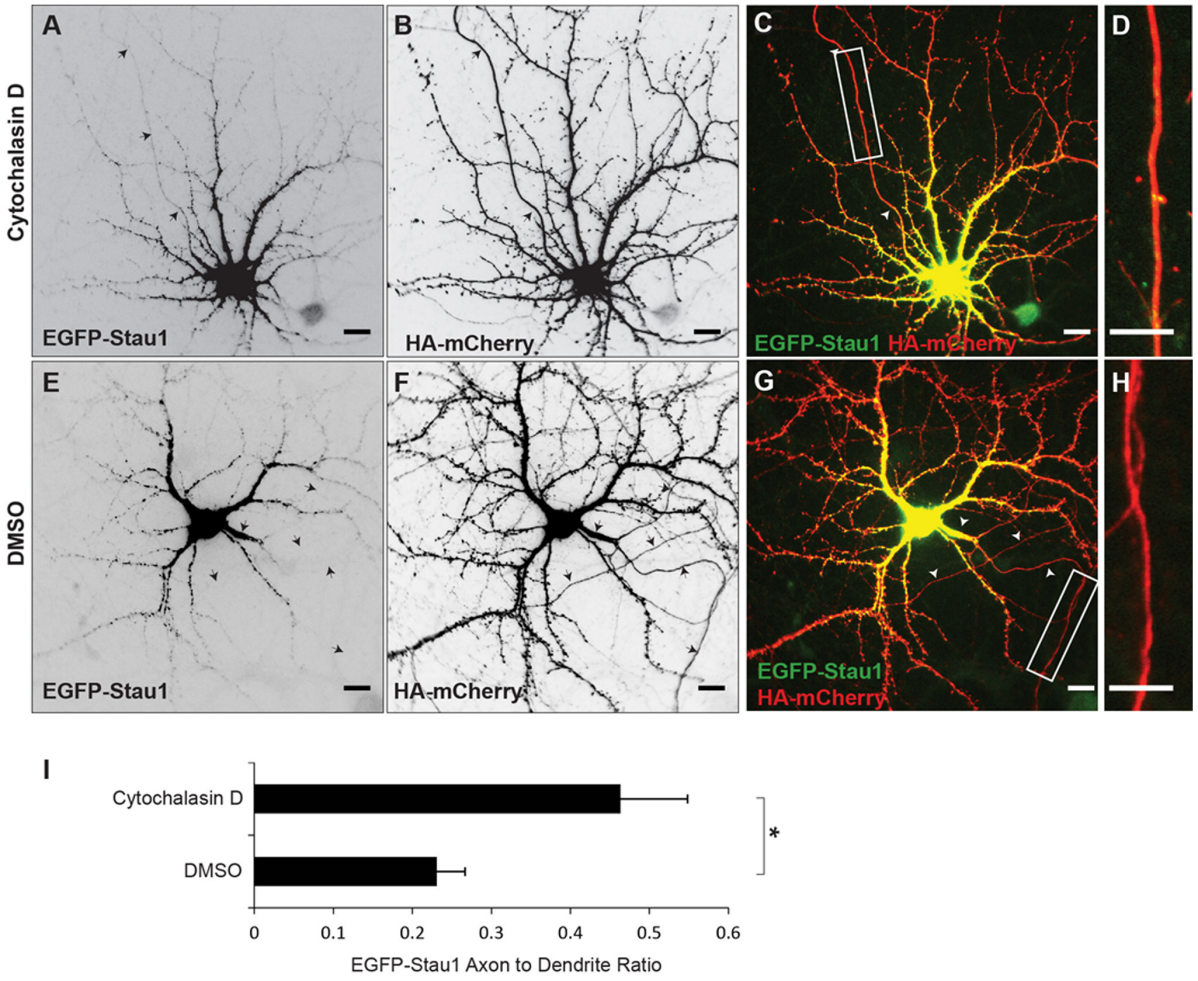
Intact actin filaments are necessary for somatodendritic localization of EGFP-Stau1. **A**. Following exposure to Cytochalasin D (4 μM) for 3 hours EGFP-Stau1 is present in the axon as well as in the dendritic compartment. **B** HA-mCherry staining in the same cell as in A. **C**. Merge of EGFP-Stau1 (green) and HA-mCherry (red). **D**. High power image of boxed area in (C). **E**. EGFP-Stau1 is absent from the axonal compartment of cortical neurons expressing HA-mCherry (**F**) and exposed to DMSO. **G**. Merge of EGFP-Stau1 (green) and HA-mCherry (red). **H**. High power image of boxed area in (G). **I**. EGFP-Stau1 axon to dendrite ratio is significantly higher in cells exposed to Cytochalasin D than in cells exposed only to DMSO. * indicates p<0.03 (Wilcoxon). Scale bar 10 μm.

**Figure 6:**
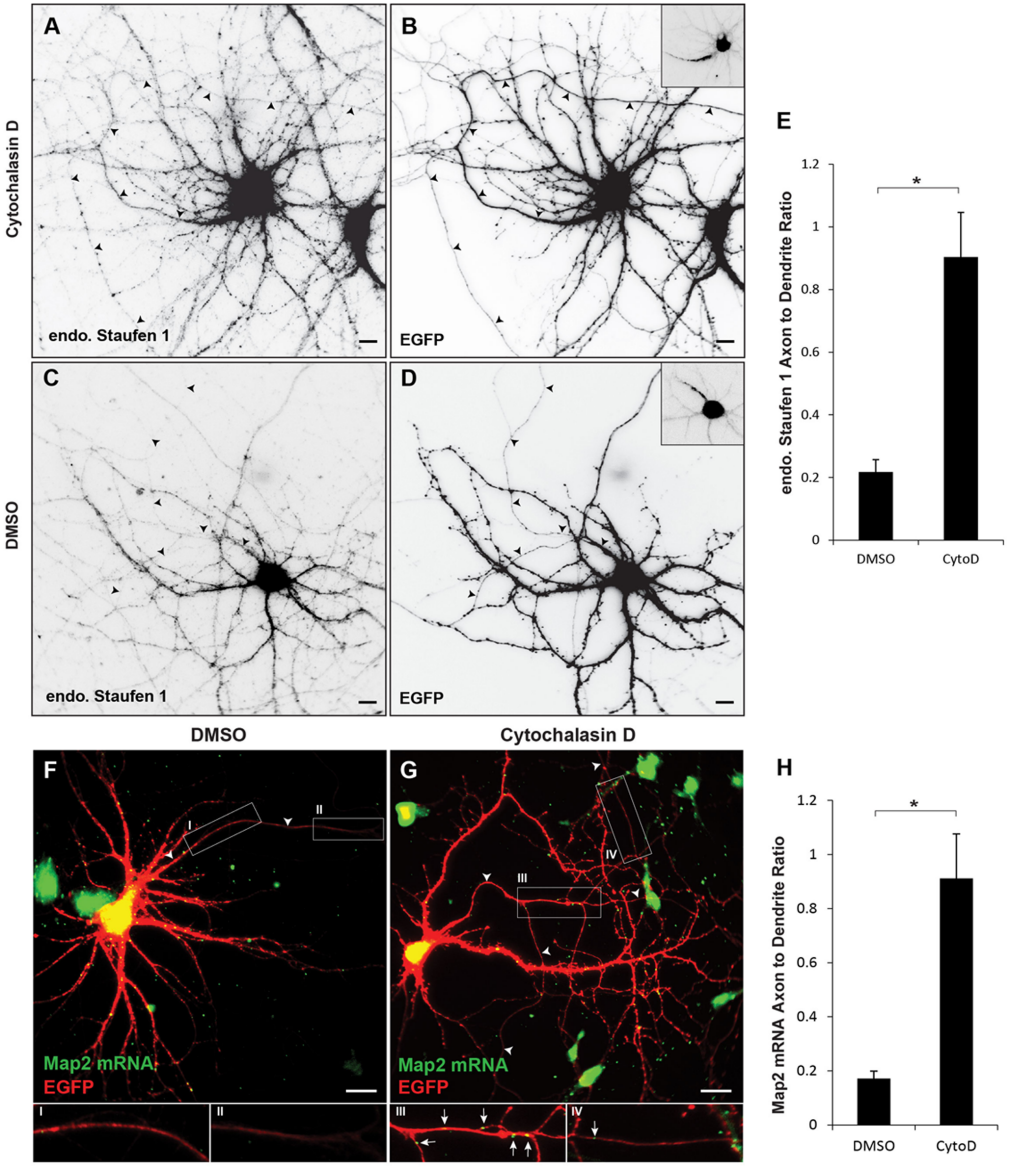
Intact actin filaments are necessary for somatodendritic localization of both endogenous Staufen 1 and Map2 mRNA. **A**. Following exposure to Cytochalasin D (4 μM) for 6 hours endogenous Staufen 1 is present in the axon as well as in the dendritic compartment. **B.** EGFP staining in the same cell as in (**A**). **C**. Endogenous Staufen 1 is absent from the axonal compartment of cortical neurons expressing EGFP (**D**) and exposed to DMSO. Insets show Ankyrin G staining, which labels the axon initial segment of the corresponding cells. **E**. The axon to dendrite ratio of endogenous Staufen 1 is significantly higher in cells exposed to Cytochalasin D than in cells exposed to DMSO alone. **F**. In a cortical neuron exposed to DMSO FISH labeling reveals Map2 expression in dendrites, but not in the axon. Insets, I and II, which are high power images corresponding to labeled boxes in (F), show a relative absence of Map2 mRNA staining in the axon. **G**. In a cortical neuron exposed to 4 μM Cytochalasin D for 6 hours Map 2 mRNA staining is present in both the axon and the somatodendritic compartment. Insets III and IV show the presence of Map2 mRNA staining in axons. **H**. Map2 mRNA axon to dendrite ratio is significantly higher in cells exposed to Cytochalasin D than in cells exposed to DMSO alone. * indicates p<0.001 (Wilcoxon). Scale bar is 10 μm.

Our finding that Myosin Va is necessary for localization of EGFP-Stau1 to the somatodendritic compartment would suggest that intact actin filaments also play a permissive role in EGFP-Stau1 localization. To test this hypothesis we depolymerized actin filaments using 4 μM Cytochalasin D in DMSO for 3–6 h following transfection of EGFP-Stau1 in dissociated cortical neurons. We found that under these circumstances EGFP-Stau1 localized to both axons and dendrites (ADR _EGFP-Stau1, CytoD_ = 0.46 +/− 0.08, n = 16; Fig. 5A-D). In contrast, in neurons exposed to DMSO, EGFP-Stau1 localized more specifically to the somatodendritic compartment (ADR_EGFP-Stau1, DMSO_ = 0.23 +/− 0.04, n = 10; Fig. 5E-H), which is significantly different from its localization in neurons exposed to Cytochalasin D (Fig. 5I; p<0.03, Wilcoxon). To further explore a possible role for actin filaments in the localization of RNPs, we examined the effect of depolymerizing actin filaments with Cytochalasin D on the localization of both endogenous Staufen 1 and endogenous Map2 mRNA. To provide a morphological marker, dissociated neurons were transfected with EGFP. They were then exposed to 4 μM Cytochalasin D for 6 hours, fixed and immunostained for endogenous Staufen 1. As with overexpressed, labeled Staufen 1, endogenous Staufen 1 mislocalized to the axonal compartment (ADR _endStau1, CytoD_ = 0.90 +/− 0.14, n = 14; Fig. 6A, B, E) following depolymerization of actin filaments. In the control case relatively little endogenous Staufen 1was present in the axonal compartment (ADR _endStau1, DMSO_ = 0.22 +/− 0.04, n = 9; Fig. 6C, D, E), which was significantly different from the control (p<0.001, Wilcoxon). Similarly, the ratio of Map2 mRNA labeled by in situ hybridization in the axon vs. in the dendrites was significantly greater in neurons exposed to 4 μM Cytochalasin D (ADR _Map2 mRNA, CytoD_ = 0.91 +/− 0.16, n = 12; Fig. 6G, H) than in control neurons (ADR _Map2 mRNA, DMSO_ = 0.17 +/− 0.03, n = 10; Fig. 6F, H; p<0.001, Wilcoxon). Thus, our data are consistent with intact actin filaments, as well as Myosin Va, being necessary for the localization to the somatodendritic compartment of RNPs containing Stau1 and Map2 mRNA. Note that phalloidin staining of cells exposed to Cytochalasin D revealed a punctate actin distribution, which confirmed that actin filaments had become depolymerized (data not shown).

### Interaction with Myosin Va is sufficient for localization of exogenous Hermes 1

**Figure 7:**
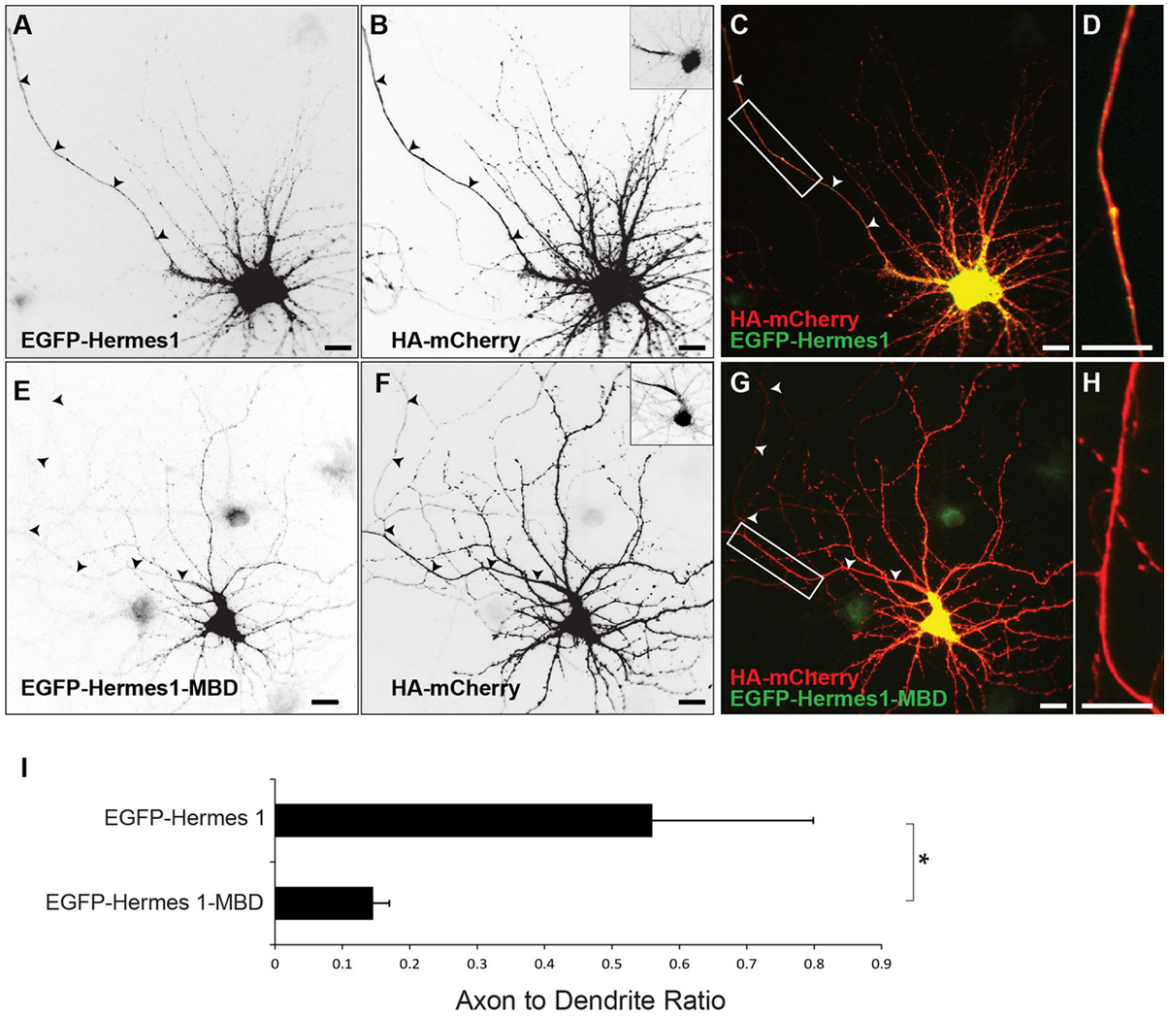
Association with Myosin Va is sufficient for somatodendritic targeting of a non-specifically localized mRNA binding protein, Hermes 1. A. EGFP-Hermes1 localizes both to the axon and dendrites of neurons co-expressing HA-mCherry (B). Insets show Ankyrin G staining, which labels the axon initial segment of the corresponding cell. C. Merge of HA-mCherry (red) and EGFP-Hermes1 (green). D. High power image of boxed area in (C). E. EGFP-Hermes1 fused to a Myosin Va binding domain (EGFP-Hermes1-MBD) localizes specifically to the somatodendritic compartment of a cortical neuron co-expressing HA-mCherry (F). G. Merge of HA-mCherry (red) and EGFP-Hermes1-MBD (green). H. High power image of boxed area in (G). I. The axon to dendrite ratio of Hermes 1 is significantly higher than that of Hermes 1-MBD. * indicates p<0.02 (Wilcoxon). Scale bar is 10 μm.

Previously, it has been shown that interaction with Myosin Va plays an instructive, as well as a permissive, role in the localization of transmembrane proteins to the somatodendritic compartment. To determine whether Myosin Va plays such a role in the localization of RNPs we investigated whether fusing a Myosin Va binding domain to Hermes 1, an mRNA binding protein that is found in both axons and dendrites [35], would be sufficient to cause it to localize specifically to the somatodendritic compartment. When expressed alone EGFP-Hermes1 localized non-specifically to both dendrites and axons of neurons co-expressing HA-mCherry (Fig. 7A-D, I; ADR _EGFP-Hermes1_ = 0.56 +/− 0.24, n = 15). In contrast, a fusion of Hermes 1 to a Myosin Va binding domain from Melanophilin [36], EGFP-Hermes1-MBD, localized more specifically to the somatodendritic compartment (Fig. 7E-I; ADR _EGFP-Hermes1-MBD_ = 0.15 +/− 0.02, n = 12; p<0.02, Wilcoxon). Thus, interaction with Myosin Va is sufficient to promote localization of non-specifically localized EGFP-Hermes1 to the somatodendritic compartment. Thus, our data suggest that interaction with Myosin Va is both necessary and sufficient to mediate localization of RNPs to the somatodendritic compartment.

## Discussion

In neurons RNPs carrying mRNA transcripts and their associated proteins are transported to specific subcellular locations, allowing temporal and spatial control over protein expression. In this paper we examine the mechanism by which neurons confine certain RNPs specifically to the somatodendritic compartment. We find that the plus end-directed myosin motor Myosin Va is necessary for localization of both exogenous and endogenous Staufen 1 and of Map2 mRNA to the somatodendritic compartment. Similarly, intact actin filaments are necessary for somatodendritic localization of both endogenous and exogenous Staufen 1 and Map2 mRNA. Finally, interaction with Myosin Va is sufficient to enhance the localization to the somatodendritic compartment of Hermes 1, an mRNA binding protein that in wild-type form is found in both axons and dendrites.

The RNPs examined here are likely transported by kinesin motors such as Kif5 and Kif3C, as Kif5 was found to be associated with Staufen and Kif3C with FMRP [21], [37]. Could it be that association with Kif5 alone is sufficient to target the RNPs to the dendrites? This is unlikely for several reasons. Firstly, numerous axonal proteins interact with Kif5 including APP, β-Secretase, and Presenilin 1 [38], [39], and thus it appears to be capable of transport to axons. Furthermore, a truncated Kif-5 that is missing its tail domain and can work autonomously is targeted very specifically to the axon [40], [41]. Indeed, it is not clear that there is a kinesin isoform that autonomously targets specifically to the somatodendritic compartment, as kinesins that are found in dendrites under wild-type conditions localize to both axons and dendrites when expressed in a constitutively active, autonomous configuration [40]. In addition, such kinesins do not appear to bind preferentially to stable microtubules, which predominate in axons or to unstable microtubules, which are found in dendrites [42].

Another motor that might contribute to the targeting of RNPs is Dynein. The role of Dynein in the subcellular localization of RNPs has mainly been studied in oocytes of D. melanogaster, [43], however, a number of results suggest that it could also be involved in polarized targeting of RNPs in neurons. For instance, the parallel alignment of microtubules in a plus end distal configuration [44] suggests that RNPs carried by Dynein would be unable to enter the axon, and thus would be localized specifically to the dendrites. This idea was confirmed by experiments where peroxisomes that were inducibly attached to dynein motors were present only in dendrites [45]. There is evidence that dynein contributes to the transport and possibly localization of RNPs in neurons, but the exact role remains uncertain. RNPs containing CPEB, and Map2 mRNA that travel to dendrites on microtubules are associated with both kinesin motors and Dynein [46]. Similarly, in drosophila S2 cells FMRP associates with both Dynein and Kinesin-1 and knockdown of either protein resulted in the failure of FMRP granules to move [47]. In addition, there is evidence that FMRP, in cooperation with the Dynein adaptor protein BicD, influences dendritic morphogenesis in the nervous system of D. melanogaster larvae [48]. Thus, a role for dynein in the localization of mRNA to dendrites is likely, and merits further investigation. However, determining whether dynein plays a specific role in targeting that is independent of its role in maintaining neuronal structure could be quite difficult, as blocking dynein function leads to dramatic changes in neuronal structure that could easily have secondary effects on the trafficking of mRNAs [49].

The results described in this paper clearly show a role for Myosin Va and actin filaments in the localization of dendritic RNPs. These results are consistent with previous work showing that Myosin Va participates in the transport and targeting of mRNAs. Notably, Myo4P, a Myosin V homolog in S. cerevisiae transports ASH1 to the bud tip in actin-dependent manner [50]. In drosophila oocytes, Myosin Va is responsible for transporting Oskar mRNA to the posterior pole [51]. Myosin Va is also responsible for intracellular mRNA distribution in primary fibroblasts [52].

Previously, it has been shown that Myosin Va and actin filaments are necessary for localization of transmembrane proteins to the somatodendritc compartment, and that interaction of these proteins with Myosin Va is sufficient to mediate somatodendritic localization [25]. Live imaging studies of vesicles carrying dendritic transmembrane proteins showed that they enter both axons and dendrites upon release from the Golgi [53]. Once in the axon, however, such vesicles halt and reverse, so that they are prevented from reaching the distal axon. Moreover, these halting and reversing events are dependent on both Myosin Va and actin. Together these results suggest a model where actin filaments in the axon initial segment (AIS) interact with myosin motors to prevent vesicles from entering the distal axon [54]. This model is also consistent with results showing that large, inert, intracellular molecules are prevented from diffusing from the cell body to the distal axon [55], and that transmembrane proteins are prevented from diffusing along the surface of the AIS [56], both in an actin dependent fashion. Furthermore, a recent study found that concentrated networks of actin filaments oriented with their plus ends facing the cell body are present in the AIS [57]. Vesicles carrying dendritic proteins halt and reverse when they come into contact with these networks, which are known as actin patches.

The experiments in this paper provide evidence that ribo-nucleoprotein particles are localized to the somatodendritic compartment through a similar actin/Myosin Va-dependent mechanism that underlies localization of transport vesicles carrying transmembrane proteins. Thus, it provides a basis for understanding the molecular mechanisms by which mRNAs are localized to specific subcellular locations in neurons and establishes that the actin/Myosin Va mechanism contributes to the polarized targeting of multiple classes of macromolecules within neurons.

## Supporting Information

Figure S1 **Labeling with sense and anti-sense In Situ Hybridization probes.** A. Cortical neuron labeled using FISH with sense probe shows negligible labeling within processes. **B**. Same neuron as in (A) labeled with EGFP. **C**. Cortical neuron labeled using FISH with anti-sense probe shows labeling in processes. **D**. Same neuron as in (C) labeled with EGFP.
(JPG)

Figure S2 **Expression of siRNA against Myosin Va for four days reduces expression of its target protein in rat cortical neurons.** **A**. Myosin Va staining in cortical neurons from a culture transfected with siRNA against Myosin Va (MyoVa siRNA). **B**. Tubulin staining in the same cells as in (A). **C**. Merge of Myosin Va staining (green) and Tubulin staining (red). Yellow indicates cells expressing both proteins, red indicates cells expressing only Tubulin. Arrows point to transfected cells. **D**. Expressed HA-mCherry, indicating cells transfected with MyoVa siRNA. Note that transfected cells have a dramatic reduction in the amount of Myosin Va present (A-D). **E**. Myosin Va staining in cortical neurons from a culture transfected with scrambled siRNA. **F**. Tubulin staining in the same cells as in (E). **G**. Merge of Myosin Va staining (green) and Tubulin staining (red). **H**. Expressed HA-mCherry, indicating cells transfected with scrambled siRNA. Note that transfected cells have no reduction in the amount of Myosin Va present (E-H). **I**. Myosin Va staining in cortical neurons from a culture cotransfected with MyoVa siRNA and a cDNA encoding Myosin Va that is impervious to siRNA (MyoVa Mut1275). **J**. Tubulin staining in the same cells as in (I). **K**. Merge of Myosin Va staining (green) and Tubulin staining (red). **L**. Expressed HA-mCherry, indicating cells transfected with Myosin Va siRNA and Myosin Va Mut 1275. Note that transfected cells have no reduction in the amount of Myosin Va present (I-L). **M**. Ratio of expression level of Myosin Va to that of Tubulin in cortical neurons that are untransfected, transfected with Myosin Va siRNA, scrambled siRNA or Myosin Va siRNA + Myosin Va Mut1275. * indicates p<0.001 (Kruskal-Wallis), ns indicates difference is not significant, (p>0.1, Kruskal-Wallis). Scale bar is 10 μm.
(JPG)
