# Supplementary figures and images for: Actin and Myosin-Dependent Localization of mRNA to Dendrites
Source: PLoS One. 2014 Mar 17;9(3):e92349. doi: 10.1371/journal.pone.0092349 (PMC3956895; doi:10.1371/journal.pone.0092349)

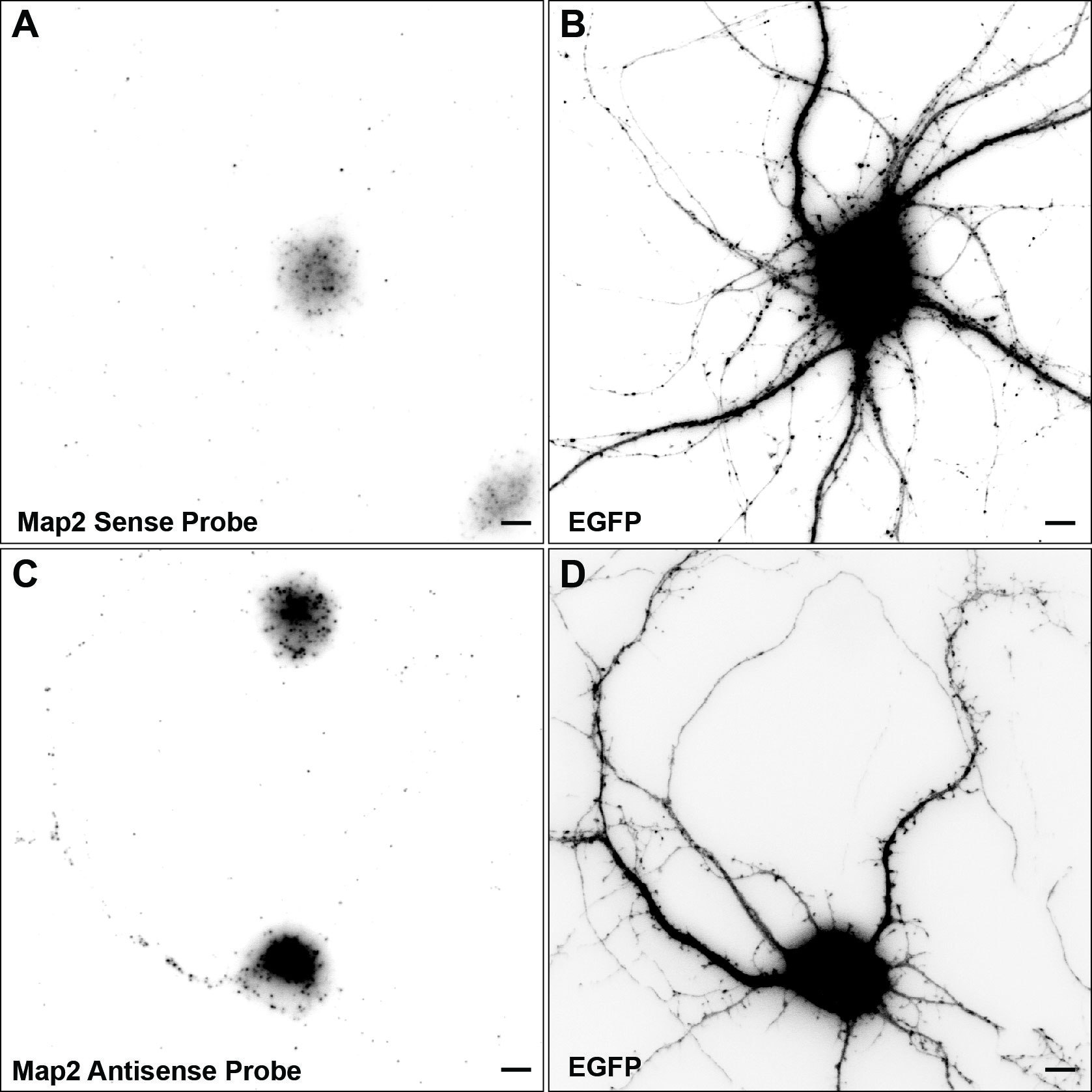

Supplement: Figure S1 — Labeling with sense and anti-sense In Situ Hybridization probes. A. Cortical neuron labeled using FISH with sense probe shows negligible labeling within processes. B. Same neuron as in (A) labeled with EGFP. C. Cortical neuron labeled using FISH with anti-sense probe shows labeling in processes. D. Same neuron as in (C) labeled with EGFP. (JPG) [file pone.0092349.s001.jpg]

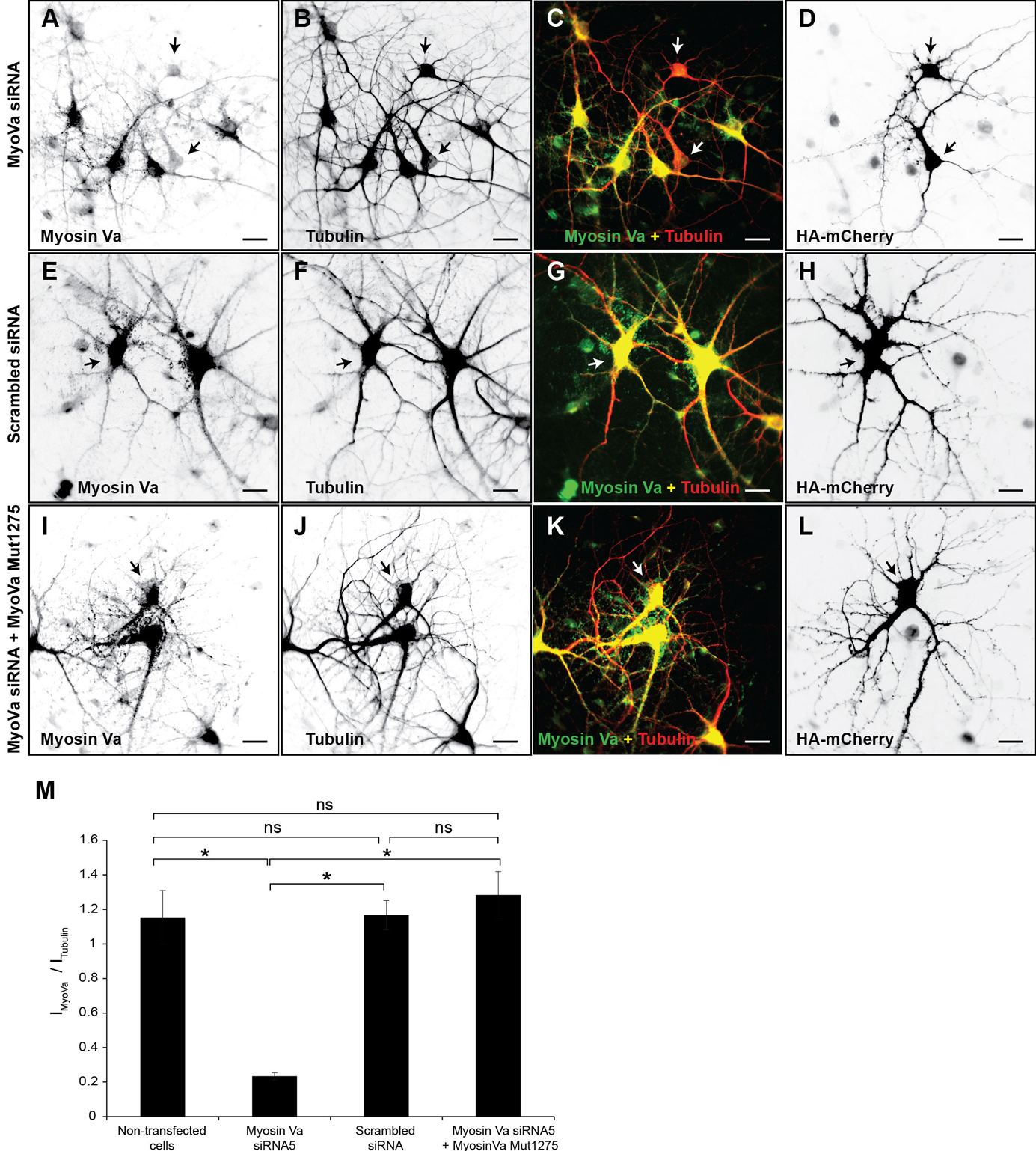

Supplement: Figure S2 — Expression of siRNA against Myosin Va for four days reduces expression of its target protein in rat cortical neurons. A. Myosin Va staining in cortical neurons from a culture transfected with siRNA against Myosin Va (MyoVa siRNA). B. Tubulin staining in the same cells as in (A). C. Merge of Myosin Va staining (green) and Tubulin staining (red). Yellow indicates cells expressing both proteins, red indicates cells expressing only Tubulin. Arrows point to transfected cells. D. Expressed HA-mCherry, indicating cells transfected with MyoVa siRNA. Note that transfected cells have a dramatic reduction in the amount of Myosin Va present (A-D). E. Myosin Va staining in cortical neurons from a culture transfected with scrambled siRNA. F. Tubulin staining in the same cells as in (E). G. Merge of Myosin Va staining (green) and Tubulin staining (red). H. Expressed HA-mCherry, indicating cells transfected with scrambled siRNA. Note that transfected cells have no reduction in the amount of Myosin Va present (E-H). I. Myosin Va staining in cortical neurons from a culture cotransfected with MyoVa siRNA and a cDNA encoding Myosin Va that is impervious to siRNA (MyoVa Mut1275). J. Tubulin staining in the same cells as in (I). K. Merge of Myosin Va staining (green) and Tubulin staining (red). L. Expressed HA-mCherry, indicating cells transfected with Myosin Va siRNA and Myosin Va Mut 1275. Note that transfected cells have no reduction in the amount of Myosin Va present (I-L). M. Ratio of expression level of Myosin Va to that of Tubulin in cortical neurons that are untransfected, transfected with Myosin Va siRNA, scrambled siRNA or Myosin Va siRNA + Myosin Va Mut1275. * indicates p<0.001 (Kruskal-Wallis), ns indicates difference is not significant, (p>0.1, Kruskal-Wallis). Scale bar is 10 μm. (JPG) [file pone.0092349.s002.jpg]
